# Reforming General Academic English: a public-health-themed curriculum for Chinese medical students

**DOI:** 10.3389/fpubh.2026.1960458

**Published:** 2026-10-28

**Authors:** Qiqi Sima, Mengyue Xu, Xiaorui Chen

**Affiliations:** 1 School of Nursing, Shandong Medical and Pharmaceutical University, Yantai, China; 2 Department of Gynecology, Jinan Maternal and Child Health Hospital, Jinan, China; 3 Department of Neurology, Shandong Medical and Pharmaceutical University Hospital, Binzhou, China; 4 School of International Studies, Shandong Medical and Pharmaceutical University, Yantai, China

**Keywords:** blended learning, Chinese medical students, curriculum reform, General Academic English, interdisciplinary teaching, process-oriented assessment, public health

## 1 Introduction

Public health is an interdisciplinary system that integrates natural science, social practice, and humanistic concepts, committed to safeguarding the population-wide health of society. The global public health crisis triggered by COVID-19 has further pushed public health education into a core position of higher medical education, and integrating public health perspectives into general courses has become a critical trend of disciplinary integration.

As a transitional bridge connecting general English and medical English for specific purposes (ESP), General Academic English (GAE) at Shandong Medical and Pharmaceutical University (SDMPU) undertakes multiple educational missions: improving students' academic English literacy, fostering autonomous learning competence (1), and meeting national talent cultivation requirements that emphasize humanistic awareness, global vision and international communication capabilities (2). Integrating public health themes into GAE serves as a targeted curriculum optimization approach, which effectively bridges medical humanities and public health expertise, and responds to China's New Liberal Arts initiative launched in 2019 (3). The initiative aims to break traditional disciplinary boundaries and promote interdisciplinary thinking to cultivate high-level compound medical talents.

After years of iterative teaching practice, the public-health-themed GAE curriculum at SDMPU has formed preliminary interdisciplinary features. Nevertheless, reflective curriculum analysis indicates that the current teaching arrangement still fails to fully achieve the intended interdisciplinary educational goals. Multiple structural, organizational and practical limitations constrain the curriculum's interdisciplinary cultivation effects. Based on long-term front-line teaching observations and a systematic review of relevant domestic and international studies on medical academic English and public health pedagogy, this paper argues that targeted curriculum reconstruction is necessary to better align GAE instruction with contemporary educational demands, and the institutional talent training positioning.

The public-health-themed GAE course examined herein is a 1.5-credit elective offered in the fourth semester for second-year undergraduate Clinical Medicine (Bachelor of Medicine—5-year program) students. Cohorts for this elective typically include approximately 700 students. The course is coordinated by one dedicated overall coordinator and delivered by five English instructors with academic backgrounds in linguistics or literature, each permanently assigned to a fixed teaching chapter; no public-health specialists participate on a regular basis. The course comprises 32 whole-class in-person lecture hours throughout the semester; small-group activities are embedded within lecture sessions, with no separate dedicated tutorial time, and self-paced pre-class Massive Open Online Courses (MOOC) materials are assigned as out-of-class preparation and are therefore excluded from the 32 contact hours. Assessment consists of one formal final examination weighting 60% and continuous assessment weighting 40%; within the continuous component, group assignments account for 20% and individual assignments for 80%. Core thematic coverage encompasses health governance, nutritional health, genetics, mental health, and traditional Chinese medicine. A concise overview of key programmatic features is provided in Table 1 for cross-institutional reference.

**Table 1:** Overview of the public-health-themed GAE course.

Item	Description
Course credit	1.5-credit elective
Offered semester	Taught in the fourth semester to second-year students
Cohort size	Approximately 700 students per cohort
Degree programme	Undergraduate clinical medicine (Bachelor of medicine—5-year program)
Course coordinator	One dedicated overall course coordinator
Instructor team	Five English instructors (linguistics/literature backgrounds); each instructor is permanently assigned to a fixed teaching chapter; no regular participation from public-health specialists
Contact hours	32 contact hours in total, all delivered in-person; pre-class MOOC materials are excluded from the contact-hour count as they are completed outside class
Teaching format	Whole-class in-person lectures throughout the 32 h; small-group activities are embedded within lecture sessions, with no separate dedicated tutorial hours; self-paced MOOC materials serve as pre-class preparation
Assessment type	One formal examination (final exam, 60%); continuous assessment (40%), of which group assignments 20% and individual assignments 80%
Core thematic topics	Health governance, nutritional health, genetics, mental health, and traditional Chinese medicine

## 2 Theoretical framework and analytical rationale

This opinion article builds its curriculum reform arguments upon three interrelated theoretical and policy foundations: ESP curriculum design principles, the interdisciplinary philosophy of China's New Liberal Arts initiative, and community-centered perspectives in public health education. In addition, it adopts a normative analytical approach guided by Kern's six-step curriculum development framework.

First, ESP principles advocate the integration of language skill training and disciplinary content learning, focusing on context-specific professional communicative needs rather than generalized language instruction (4). Second, the New Liberal Arts initiative promotes cross-disciplinary integration to cultivate compound talents capable of coping with complex social and public health challenges (5). Third, community-centered public health education emphasizes scenario-based practice and real-world problem-solving, encouraging classroom knowledge transfer into authentic community health contexts (6).

These three foundations complement and reinforce one another: ESP provides the fundamental language-teaching paradigm; the New Liberal Arts offers macro-level interdisciplinary policy guidance; and community-centered public-health education delivers practice-oriented instructional principles. Collectively, these theoretical underpinnings support the curriculum reconstruction strategies proposed in Section 4, including student-centered teaching philosophy, interdisciplinary teaching-team construction, project-driven blended-learning arrangements, and multi-dimensional evaluation mechanisms.

The analysis adopts a normative logical framework that identifies practical teaching challenges, diagnoses existing curriculum dilemmas, develops reform-oriented design concepts, and proposes feasible implementation pathways. The arguments are synthesized from published pedagogical literature, national educational policy documents (7), and long-term front-line teaching observations from GAE English educators at SDMPU. No original questionnaire surveys, student sampling, interviews, or statistical analyses have been conducted for this manuscript, and no primary empirical data have been generated. All reflections on curriculum limitations are generalized from published educational research and routine classroom teaching practice.

Kern's six-step curriculum development framework consists of six sequential stages: problem identification and general needs assessment, targeted needs assessment, goals and objectives, educational strategies, implementation, and evaluation and feedback. Drawing on this well-established framework for health professions education (8), this opinion article conducts practical curriculum needs diagnosis and proposes corresponding curriculum goals, instructional strategies, implementation pathways, and evaluation suggestions.

To clarify, this opinion article analyses an existing course for iterative improvement instead of presenting a *de-novo* full-cycle curriculum-development project. We therefore treat Kern's framework as an informing reference rather than the strict organizational backbone of the manuscript. We adopt the current structure (curriculum characteristics-dilemmas-reconstruction pathways), which better fits our analytical focus on describing the course status quo and diagnosing implementation-related challenges. Key elements from Kern's steps are discussed across corresponding sections, while full iterative execution of the six-step cycle is reserved for future practical curriculum delivery.

Beyond these overarching foundations, three supplementary analytical lenses are adopted for targeted analysis in subsequent sections. Self-determination theory (SDT) provides a framework to examine learner motivation and barriers to engagement. The community of inquiry (CoI) framework guides the design of AI-enhanced interactive learning activities. Programmatic Assessment underpins the multi-stakeholder, process-oriented evaluation system proposed for curriculum reconstruction. These three lenses serve as specialized analytical tools embedded within the broader Kern-guided curriculum analysis. The interrelationships among these theoretical foundations and analytical lenses are visualized in Figure 1.

**Figure 1:**
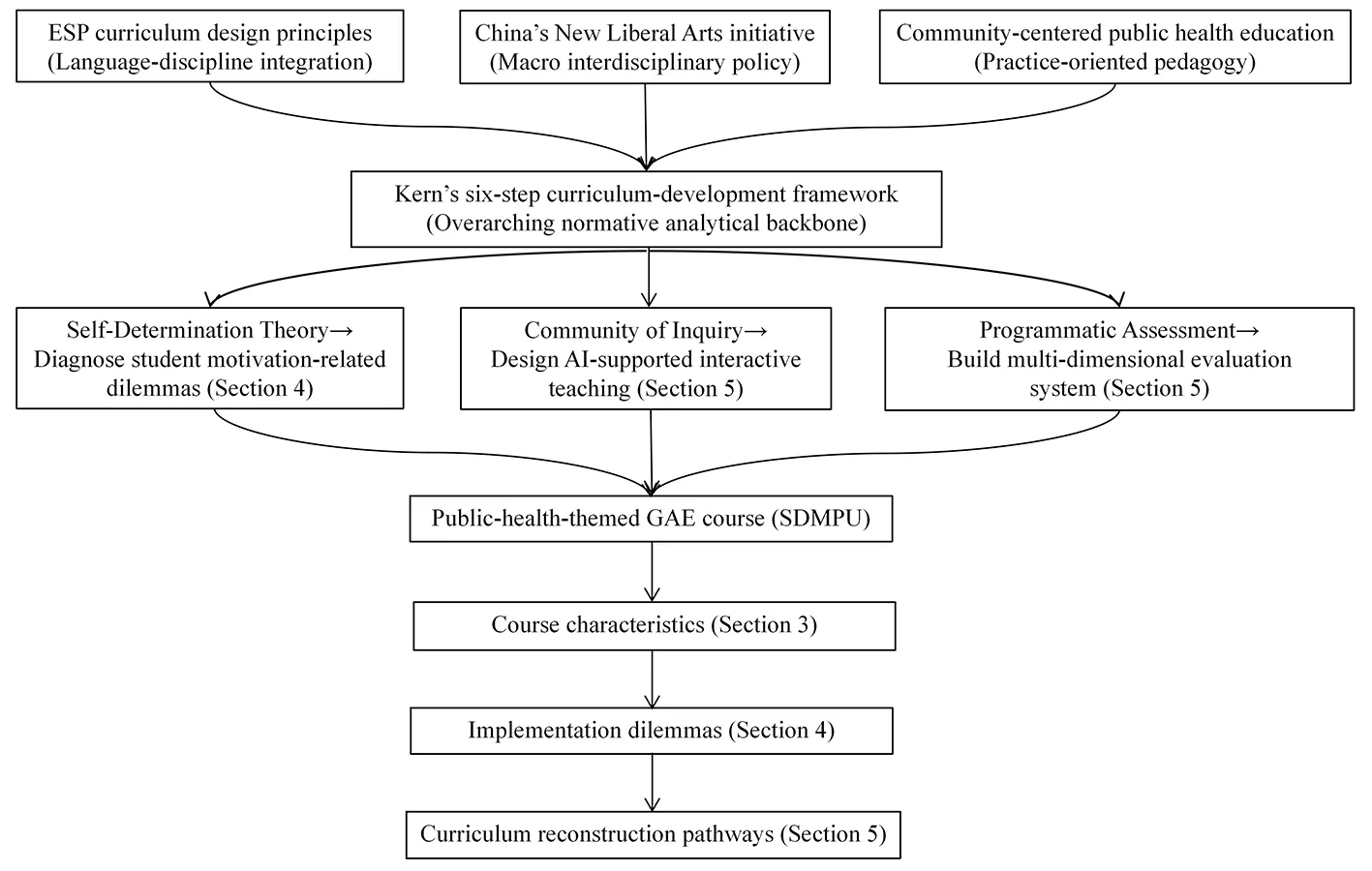
Conceptual framework for analyzing the public-health-themed GAE course, integrating multiple educational theories to guide sequential analysis of course characteristics, implementation dilemmas and reconstruction pathways.

## 3 Core characteristics of public-health-themed GAE at SDMPU

The characteristics outlined in this section describe the intended formal curriculum design of the public-health-themed GAE course, representing its structural and thematic aspirations. This section addresses four core attributes: interdisciplinary integration, popular science-oriented curriculum design, diversified blended learning arrangements, and cultivation of cross-cultural critical thinking. Nevertheless, gaps remain between curricular design intentions and day-to-day instructional delivery; these practical implementation challenges are analyzed further in Section 4.

The public health-oriented GAE curriculum transcends the narrow boundaries of conventional language teaching and integrates interdisciplinary knowledge across public health, clinical nutrition, basic medical sciences, genetics, psychology and medical humanities. Different from general English courses that focus merely on linguistic competence, this interdisciplinary embedding enriches students' systematic knowledge of public health and fosters their multidimensional cross-disciplinary thinking, enabling learners to interpret medical and public health issues from multiple perspectives beyond pure language acquisition.

The textbook system of this GAE course at SDMPU takes public health popularization as the main thread throughout curriculum design. It covers a wide range of authentic and practical public health themes, including disciplinary origin debates, cutting-edge medical technologies, and prevalent public health challenges such as dietary-related diseases, nutritional imbalance, and mental health disorders. Key professional public health vocabulary is annotated and interpreted in the textbook, taking into account the dual attributes of language foundation and popular science dissemination. This design aligns with the positioning of GAE as a transitional bridge course between general English and specialized medical English.

In terms of teaching organization and implementation, the GAE course adopts an online-offline blended learning model supported by a professional MOOC platform, which realizes full-cycle teaching design covering pre-class preview, in-class thematic expansion, and after-class knowledge consolidation. The course is jointly delivered by a professional teaching team, who adopt differentiated and diversified instructional strategies according to different thematic chapters. Multiple teaching forms, including traditional classroom lectures, flipped classroom tasks, thematic group presentations, and collaborative academic workshops, are flexibly applied in teaching practice. Such diversified blended learning designs seek to enrich classroom teaching scenarios, stimulate students' learning initiative, and enhance learners' proficiency in public health English.

In curriculum content arrangement, comparative reading materials are adopted to trigger critical thinking regarding transnational public health governance. For example, thematically paired English texts concerning Chinese and American healthcare systems are introduced in routine teaching. Such comparative reading enables medical students to intuitively analyze institutional differences in public health governance across countries, reflect on the practical dilemmas and optimization directions in global public health development, and integrate linguistic training with humanistic and international public health cognition. This instructional design helps students avoid simplistic, stereotyped perceptions of global public health issues and gradually develop objective, critical and globalized public health literacy. These comparative reading activities aim to foster students' intercultural communicative competence (9) and cultural humility (10), which constitute core dimensions of established global health competency frameworks. Beyond improving basic academic language proficiency, such comparative materials encourage medical students to recognize diversified international public health contexts, embrace cross-cultural differences, and build global-minded perspectives for future professional cross-cultural health communication.

## 4 Typical dilemmas restricting the effectiveness of the current GAE curriculum

While the GAE course possesses promising design-related characteristics laid out in Section 3, gaps between formal curriculum intentions and practical instructional delivery give rise to multiple implementation-level dilemmas that restrict the expected interdisciplinary teaching effectiveness. This section examines these practical challenges across four dimensions: insufficient learning and teaching motivation, fragmented team teaching and overloaded group assignments, disjointed blended learning, and limited social practice opportunities.

At SDMPU, General Academic English is set as an elective course, which brings structural challenges for learning motivation. As documented in existing pedagogical studies (11), elective-course status may shape learners' course-selection priorities. From the authors' front-line teaching observations, some students may prioritize credit completion when selecting electives and may allocate comparatively less time and energy to GAE compared with compulsory medical majors, with superficial engagement in homework and classroom tasks observable among a subset of learners. This practical concern resonates with the core argument from SDT, which highlights how contextual factors and motivational regulation shape learners' perceived autonomy and quality of engagement (12). The elective attribute may also create challenges for consistent teaching standards. Formative evaluation relies heavily on group assignments lacking standardized supervision mechanisms. Based on the authors' teaching observations together with published literature, free-riding behaviors can occasionally emerge within learning teams (13), which may reduce the expected training benefits of collaborative tasks.

The rotational team teaching model, in which different instructors are responsible for separate chapters, may bring notable practical trade-offs despite diversifying classroom practice. From the authors' front-line teaching observations, frequent instructor handovers can lead to fractured logical coherence across chapters and inconsistent instructional rhythms, imposing ongoing adaptation pressure on students exposed to divergent teaching styles. Meanwhile, over-reliance on group assignments may add substantial workload for medical students, who are already occupied by intensive professional coursework. As also highlighted in existing relevant literature (13), poorly designed group-based tasks may create conditions raising potential risks of academic misconduct including plagiarism of group assignments.

Although flipped classroom and MOOC resources are formally incorporated, the overall teaching logic still leans toward traditional offline lecture-centered models, which may limit the complementary value of online resources. Common practical problems include outdated, low-matching MOOC materials disconnected from classroom themes, and most instructors take offline teaching as the absolute core while online resources only serve as superficial add-ons without systematic integrated design (14). The separation of online knowledge input and offline practical training can render the blended-learning framework partially nominal in practice.

Public health education emphasizes community participation and real-scene problem-solving, enabling students to apply disciplinary vocabulary and logical thinking to solve real social health challenges (15). However, constrained by traditional classroom-centered teaching concepts, the practical activities of the current GAE course at SDMPU are limited to campus-based tasks such as poster design. There is a lack of extended off-campus social practice targeting community residents, foreigners and public health service institutions. The disconnection between classroom theoretical learning and real public health scenarios may create a notable gap between curriculum design goals and practical teaching outcomes.

## 5 Reconstruction paths of public-health-themed GAE curriculum

Building on the curriculum dilemmas diagnosed in Section 4, this section outlines multi-faceted reconstruction pathways for the public-health-themed GAE course. The reform proposals cover four interconnected dimensions: revised teaching philosophy, interdisciplinary teaching-team development, project-driven blended-learning design, and multi-dimensional process-oriented evaluation.

Following Step 3 (goals and objectives) of Kern's curriculum-development framework, this paper defines three categories of learning objectives tailored to this public-health-themed GAE curriculum, informed by ESP principles and global-health competency frameworks. Linguistic objectives require students to comprehend public-health-related English texts and construct coherent written and oral academic expressions concerning basic public health topics. Interdisciplinary cognitive objectives enable learners to interpret selected public health issues from integrated medical and humanistic perspectives. Cross-cultural competence objectives require students to exercise cultural humility and global-health awareness to engage in respectful cross-cultural communication on health-related challenges.

To achieve these learning objectives, curriculum content can be enriched with interdisciplinary resources. Rooted in the multi-dimensional integration logic of public health education covering biomedicine, social science, environmental governance and medical ethics (16), GAE may seek to expand curriculum content with cross-domain resources such as medical resource allocation, occupational health and environmental public health, and embed historical humanistic materials to enrich thematic depth.

Two core transformation directions may be identified in curriculum positioning: shifting from single discipline-oriented language training to social demand-oriented interdisciplinary talent cultivation (17), and integrating ideological and political education throughout all thematic modules. Teachers can design diversified off-campus practical activities closely linked to public health themes: producing English popular science videos, delivering community public health lectures, participating in Model United Nations debates on global health, and surveying the application of English public health signs in local medical institutions. These practices may help strengthen medical students' social responsibility and support the fundamental educational goal of fostering virtue through teaching (18, 19).

As a fundamental institutional guarantee for constructing a stable interdisciplinary blended learning system, SDMPU may consider upgrading GAE to a compulsory course to mitigate motivational deficits originating from the elective-course positioning. Such institutional adjustment may elevate the course's institutional status, help raise students' awareness of the course, and enhance instructors' teaching dedication. With improved curriculum positioning, both students and teaching staff may maintain more sustained and effective engagement, helping lay a solid institutional foundation for standardized, high-quality interdisciplinary blended teaching and public health English training.

To address instructional fragmentation caused by frequent instructor handovers in rotational team teaching, SDMPU may establish a fixed core teaching team to unify instructional logic, textbook standards and assessment requirements, thereby reducing repeated adaptation burdens for students. In response to the interdisciplinary requirements of the New Liberal Arts initiative, medical English instructors and clinical and public health specialists can form long-term collaborative teaching communities. Professional physicians deliver instruction on core public health thematic knowledge, while language instructors focus on academic expression, poster composition and international communication skills. Drawing on mature interdisciplinary teaching practices from Capital Medical University (20), this dual-instructor collaborative model may support both professional accuracy and satisfactory language training outcomes (21).

To restructure the nominal blended-learning model, the proposed curriculum adopts a project-driven blended-learning design grounded in the CoI framework, which conceptualizes effective online-offline education through three interrelated presences: teaching presence, social presence, and cognitive presence (22). Within the GAE curriculum, pre-class MOOC learning supports individual knowledge acquisition; in-class project-oriented, public-health-themed tasks foster peer interaction and collaborative inquiry, while targeted instructor guidance sustains coherent teaching presence throughout the whole learning cycle.

The blended learning system realizes rational functional division and organic coordination between online and offline instructional links: online MOOC platforms support pre-class theoretical learning, case reading, and autonomous knowledge sorting (23), while offline classroom sessions focus on project-based practical tasks centered on authentic public health themes (24). Taking the “Diet and Health” chapter as an example, instructors can design diversified group projects oriented to public health science communication (25). Specific tasks include producing bilingual health popular science videos (26), designing publicity posters for foreign-resident communities, and participating in themed international debate activities (27). To optimize learning outcomes, instructors may moderately adjust the proportion of group assignments and individual tasks to balance student workload. Such flexible arrangement helps alleviate excessive academic pressure on medical students and mitigate potential risks of superficial learning and academic misconduct. Through systematic online-offline integration and well-structured project design, students are able to apply academic English competencies in authentic public health communication scenarios, gradually cultivating the international communication capacity required for future participation in global health governance.

Additionally, widely accessible generative AI translation tools bring new pedagogical considerations to this English teaching context. Although AI translation offers convenient linguistic support for medical students, over-reliance on machine translation may hinder vocabulary retention and the development of independent English expression (28). In this GAE curriculum, instructors may adopt balanced strategies: designing offline output tasks without translation tools, prioritizing active language production in authentic public health communication activities, and embedding guidance on proper AI tool use within teaching. These measures seek to frame AI as an auxiliary aid rather than a replacement for learners' language-skill development.

Aligned with the blended learning arrangement, the curriculum evaluation system seeks to move beyond sole reliance on final examinations to establish a multi-dimensional evaluation mechanism. This multi-dimensional design aligns with the core spirit of programmatic assessment, which advocates continuous, holistic collection of learner evidence rather than high-stakes single-examination outcomes (29). First, weights for online and offline assessments may be rationally allocated (30): online platforms leverage big data to record students' autonomous learning trajectories for formative assessment (31), while offline evaluation centers on classroom engagement, group project progress and practical performance. Second, targeted evaluation criteria may be developed for public health practice activities. Relevant references include mature indicator frameworks for medical popular science video assessment (32) and standardized English public signs in hospitals (33); alternatively, task-specific evaluation dimensions can be developed according to the features of each project task. Third, expand evaluation participants to form a five-dimensional evaluation matrix covering intelligent learning platforms, student self-assessment, instructor scoring, peer review, and social feedback from practice recipients. With instructor scoring as the core benchmark, standardized peer review and post-practice written reflection could be designated as compulsory components to support comprehensive tracking of students' holistic development.

## 6 Conclusion

Despite the proposed reconstruction pathways, the large-scale curriculum reform still faces inherent institutional constraints. The large intake of approximately 700 students per cohort, together with fixed teaching hour arrangements, limits the refinement of personalized guidance and high-intensity practical training in project-based learning. Furthermore, sustainable interdisciplinary teaching relies heavily on cross-departmental incentive mechanisms. The lack of unified workload recognition and systematic teacher training may hinder the long-term stability and iterative improvement of the reconstructed curriculum system.

Even so, curriculum innovation in this direction remains meaningful. Against the backdrop of the ongoing New Liberal Arts reform, public health education has become an indispensable component of high-level medical talent cultivation. Public-health-themed General Academic English may take on the critical mission of integrating language education with interdisciplinary medical literacy cultivation.

To address the multiple practical dilemmas embedded in the current GAE curriculum, medical universities may fully integrate digital information technology into curriculum construction, enrich updated MOOC resources, and establish specialized digital databases for public health English teaching. As a transitional disciplinary language course, GAE may seek to break the limitations of traditional teacher-centered classrooms, strengthen interdisciplinary docking with public health professional training, and design diversified off-campus social practice activities. Supported by stable interdisciplinary teaching teams, project-oriented online-offline blended learning, and a multi-dimensional process-based evaluation system, the curriculum has potential to develop toward a systematic and closed-loop reform ecosystem.

As an opinion-based study, this paper proposes targeted reconstruction strategies based on prevalent teaching phenomena in medical universities, without self-collected empirical data collection. The reconstructed curriculum framework may offer theoretical references and practical implications for peer medical universities conducting public health-oriented academic English teaching reform, and could help advance the cultivation of interdisciplinary medical talents with global vision and comprehensive public health literacy.
